# P300-mediated NEDD4 acetylation drives ebolavirus VP40 egress by enhancing NEDD4 ligase activity

**DOI:** 10.1371/journal.ppat.1009616

**Published:** 2021-06-10

**Authors:** Linliang Zhang, Shixiong Zhou, Majuan Chen, Jie Yan, Yi Yang, Linjuan Wu, Dongning Jin, Lei Yin, Mingzhou Chen, Yali Qin

**Affiliations:** State Key Laboratory of Virology and Modern Virology Research Center, College of Life Sciences, Wuhan University, Wuhan, China; University of Pittsburgh, UNITED STATES

## Abstract

The final stage of Ebola virus (EBOV) replication is budding from host cells, where the matrix protein VP40 is essential for driving this process. Many post-translational modifications such as ubiquitination are involved in VP40 egress, but acetylation has not been studied yet. Here, we characterize NEDD4 is acetylated at a conserved Lys667 mediated by the acetyltransferase P300 which drives VP40 egress process. Importantly, P300-mediated NEDD4 acetylation promotes NEDD4-VP40 interaction which enhances NEDD4 E3 ligase activity and is essential for the activation of VP40 ubiquitination and subsequent egress. Finally, we find that Zaire ebolavirus production is dramatically reduced in P300 knockout cell lines, suggesting that P300-mediated NEDD4 acetylation may have a physiological effect on Ebola virus life cycle. Thus, our study identifies an acetylation-dependent regulatory mechanism that governs VP40 ubiquitination and provides insights into how acetylation controls EBOV VP40 egress.

## Author summary

Ebola virus (EBOV) is one of the deadliest pathogens, causing fatal hemorrhagic fever diseases in humans and primates. In this study, we find that P300-mediated NEDD4 acetylation facilitates EBOV egress. Acetylation promotes NEDD4-VP40 interactions which enhances NEDD4 E3 ligase activity and is essential for the activation of VP40 ubiquitination and subsequent egress. This study implies that inhibitory effect of acetylation can be regarded as an attractive candidate of drug target for the treatment of Ebola virus disease.

## Introduction

Ebola virus (EBOV) is one of the deadliest pathogens, causing fatal hemorrhagic fever diseases in humans and primates [1,2]. The outbreak in the Democratic Republic of Congo during June 2020 caused significant alarm. The core nucleocapsid structure of EBOV is composed of viral RNA, nucleoprotein (NP), viral protein (VP) 35, VP30, and the polymerase L [3–5]. Surrounding the core structure are the matrix protein VP40 and a host-derived lipid envelope where the glycoprotein (GP) is inserted [6,7].

The final process of EBOV replication is the budding of viral particles from the host cells where the matrix protein VP40 is necessary and sufficient to drive it [8–12]. VP40 connects the viral nucleocapsid to the inside of the cell membrane and promotes the egress event to achieve the whole viral life cycle, which depend on its oligomerisation ability [13]. When the C-terminal domain (CTD) of VP40 attaches to cell membranes [14], it then forms polymers through self-interactions of N-terminal domain (NTD), which contributes to the release of EBOV particles [15]. VP40 can form linear hexamers and octamer rings [16]: the hexameric VP40 plays a great role in lipid raft and viral particle formation [17,18]; the octameric VP40 is essential for binding and viral life cycle [8,19]. Although VP40 is the core driving force in EBOV egress, other viral proteins, such as GP and NP, can also enhance efficient viral particles release [20]. In addition to viral proteins for the budding process, EBOV has further developed several strategies to manipulate host proteins for mediating viral egress. For example, EBOV hijacks the host cytoskeleton and the ESCRT machinery to facilitate the assembly and budding of their virions [21–24].

The NTD of VP40 contains two overlapping late budding domains (L domains), namely _7_PTAP_10_ and _10_PPEY_13_ [5,9,25,26] and the L domains can interact with host factors containing WW domains. This interaction can make VP40 tagged post-translational modifications (PTMs), especially ubiquitination [9,27–32]. Ubiquitin functions as an internalization signal that delivers the EBOV VP40 to the cell membrane, followed by viral assembly and budding [33,34]. NEDD4 is a member of the HECT family of WW-domain- containing ubiquitin ligases that catalyzes VP40 ubiquitination which facilitates VP40 egress [9,26,30,35]. Strikingly, the expression of cellular IFN-stimulated gene *ISG15* decreases NEDD4-mediated VP40 ubiquitination. ISG15 competitively interacts with NEDD4 to block its E3 ligase activity and then inhibits EBOV VP40 egress [36,37], which provides a snapshot of the negative regulatory landscape of NEDD4-mediated VP40 budding process.

In addition to ubiquitination, a growing number of other PTMs have also been reported to function during the release of EBOV VP40 VLPs, such as phosphorylation and SUMOylation. For example, the expression of c-Abl1 stimulates the phosphorylation of VP40, which increases the release of EBOV VLPs [38]. Furthermore, VP40 also undergoes small ubiquitin-like modifier (SUMO) modification, which regulates the stability of VP40 and affects its budding [39]. Though the role of PTMs in regulating EBOV VP40 budding is well established, little is known about the acetylation controlling the budding process of VP40.

Acetylation has been identified as an evolutionarily conserved modification in key cellular processes and has recently been exploited to play a broad and critical role in viral infection [40,41]. Viruses have also evolved to manipulate the acetylation network to facilitate viral propagation at multiple steps of viral life cycle. For example, acetylation is critical in the early stages of human immunodeficiency virus type 1 (HIV-1) infections [42–44]. Likewise, Kaposi’s sarcoma-associated herpesvirus (KSHV) or human herpesvirus 8 (HHV-8) infections induce the acetylation of microtubules and facilitate subsequent transport of viral particles [45,46]. In the context of the acetylation and viral release stage, influenza A virus (IAV) nucleoprotein at K229 acetylation impairs the release of viral particles [47]. Conversely, acetylated microtubules can promote IAV release via upregulating the trafficking of viral components to the plasma membrane [48].

In this study, we found that NEDD4 was acetylated by P300 and enhancements in NEDD4 acetylation status dramatically increased NEDD4 E3 ligase activity and ubiquitination of VP40, resulting in subsequent migration of VP40 to the plasma membrane and egress. This study revealed crosstalk between acetylation and ubiquitination, which vastly expanded the functional diversity of NEDD4 in the regulation of EBOV VP40 egress process.

## Results

### Acetylation-mimicking mutation of NEDD4 promotes VP40 budding

**Fig 1:**
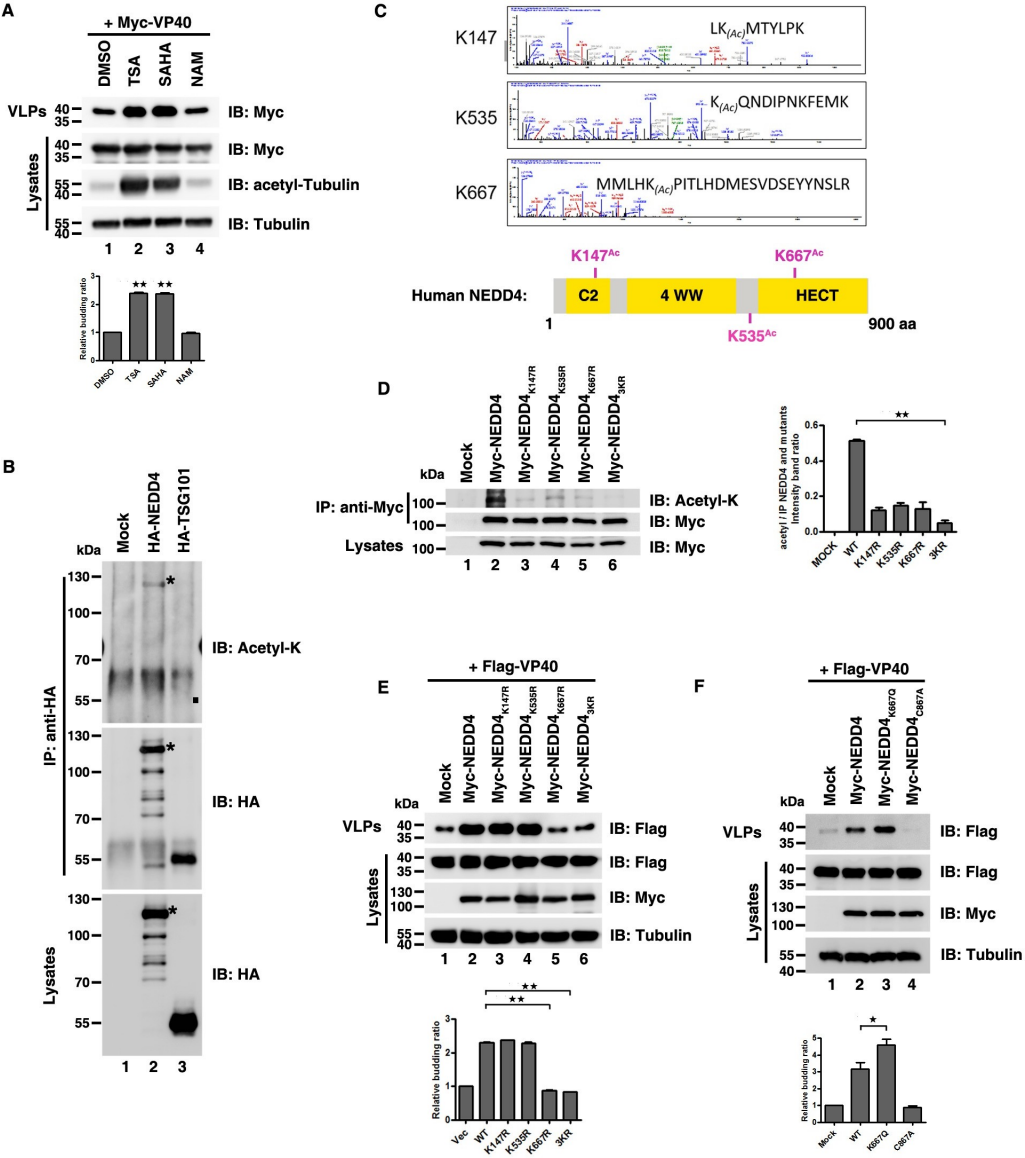
Acetylation-mimicking mutation of NEDD4 promotes VP40 budding. (A) HEK293T cells were transfected with Myc-VP40 for 40 hours and then treated with TSA, SAHA, and NAM for 8 hours and analyzed using the virus-like particle (VLP) assay. (B) HEK293T cells were transfected with indicated plasmids, immunoprecipitated with an anti-HA antibody and analyzed via immunoblotting with an anti-Acetyl-K antibody to detect the acetylation of NEDD4 and TSG101, where “*” indicated the specific NEDD4 and acetylation of NEDD4 and “■” indicated the location of the acetylation of TSG101. (C) NEDD4 acetylation sites were identified using mass spectrometry. Schematic diagram of the location of NEDD4 acetylation sites. (D) HEK293T cells were transfected with indicated plasmids, immunoprecipitated with an anti-Myc antibody and analyzed via immunoblotting with an anti-Acetyl-K antibody to detect the acetylation of the NEDD4 wild-type (WT) and mutants. (E)–(F) HEK293T cells were transfected with indicated plasmid combinations to detect the influence on the egress of VP40. Error bars, mean ± SD of three experiments. Student’s t test; *p < 0.05; **p < 0.01; ***p < 0.001.

PTMs play critical roles in the EBOV VP40 budding process [33], but protein acetylation has not yet been explored. Therefore, we aimed to investigate whether acetylation was involved in regulation of the EBOV VP40 budding process. We used three broad-spectrum deacetylase inhibitors to increase the level of cellular acetylation and then examined the release of VP40 VLPs. We found that when all proteins were detected at equivalent levels in cell lysates, the HDAC deacetylase family inhibitors trichostatin A (TSA) (Fig 1A, lane 2) and suberoylanilide hydroxamic acid (SAHA) (Fig 1A, lane 3) could facilitate the release of VP40 VLPs (compared to Fig 1A, lane 1), while the increased levels of acetyl-tubulin implied the two inhibitors had worked (Fig 1A). Interestingly, the SIRT deacetylase family inhibitor nicotinamide (NAM) could not enhance VP40 egress which suggested SIRT deacetylases may have no influence on VP40 budding (Fig 1A, compared with lanes 1 and 4). In order to verify the specificity of the enhancement on VP40 egress by HDAC deacetylase inhibitors, we treated HEK293T cells with increasing concentration of TSA. We found that VP40 VLP production was specifically increased in response to increases in TSA concentration (S1A Fig). The above data indicated that the enhanced cellular acetylation by HDAC deacetylase inhibitors may promote EBOV VP40 egress.

Given that VP40 is essential for driving viral budding, we assumed that enhanced cellular acetylation may increase VP40 acetylation levels which subsequently facilitated VP40 egress. In S1B Fig, we used an anti-acetyl-K antibody to immunoprecipitate acetylated VP40 detected via immunoblotting with an anti-Myc antibody. Consistent with our hypothesis, we found the form of VP40 acetylation in cells (S1B Fig, lane 2) and VP40 acetylation could be enhanced by TSA (S1B Fig, lane 3). Then we questioned whether VP40 acetylation could facilitate the release of VP40 VLPs. To answer the question, we needed to identify the acetylated site(s) of VP40 and confirm the acetylated site(s) had influences on VP40 egress. During the course of our study, another study reported that Zaire ebolavirus VP40 can be acetylated at numerous lysines, namely K221, K224, K225, K274 and K275 [49]. All the five sites are located in the C-terminal domain of VP40 and are essential for interactions with cell membranes [14]. Therefore, we mutated each of these K residues according to their reported sites to an arginine (R), which abolished capacity for acetylation at these positions [50], and then determined whether this lack of acetylation might affect the release of VP40 VLPs. The mutational results showed that substitution with non-acetylabtable arginine did not affect the release of VP40 VLPs compared to the wild-type (WT) VP40 (S1C Fig), suggesting that the acetylations of VP40 K residues were not involved in regulating the release of VP40 VLPs in HEK293T cells.

Since the acetylation of VP40 did not directly affect the function of its budding, we hypothesized that the acetylation of host cellular factors might regulate the budding of VP40. To test this hypothesis, we examined the acetylation of two key cellular factors, NEDD4 (an E3 ubiquitin ligase that is most widely studied in the context of VP40 budding) [9,26,30,35] and TSG101 [a key protein of the endosomal sorting complex required for transport (ESCRT) pathway that drives Ebola virus egress] [24,35,51]. Interestingly, we found that NEDD4 could be acetylated via immunoprecipitation, but TSG101 could not (Fig 1B). Furthermore, we also estimated NEDD4 acetylation sites by performing liquid chromatography-mass spectrometry (LC-MS) assays in HEK293T cells and identified three lysine acetylation sites: K147, K535 and K667 (Fig 1C). K147 is in the N-terminal C2-domain, while K667 is in the C-terminal HECT-domain (Fig 1C). Both K147 and K667 are conserved across species (S1D Fig). Moreover, the effects of the three acetylated sites on the acetylation of NEDD4 were confirmed via mutation analysis (Fig 1D). When we mutated each of these K residues to R (NEDD4_K147R_, Fig 1D, lane 3; NEDD4_K535R,_ Fig 1D, lane 4; and NEDD4_K667R,_ Fig 1D, lane 5), the acetylation level of these mutants was declined compared to WT NEDD4 (Fig 1D, lane 2). Besides, NEDD4 acetylation was blocked when three K residues were mutated to R at the same time (NEDD4_3KR;_ Fig 1D, lane 6) which further implied the specificity of NEDD4 acetylation sites.

Next, we sought to know whether these three acetylation sites of NEDD4 play a role in VP40 budding. We transfected HEK293T cells with VP40 plus WT NEDD4 and its mutants and found that NEDD4_K667R_ (Fig 1E, lane 5) and NEDD4_3KR_ (Fig 1E, lane 6) dramatically reduced VP40 VLP production, whereas NEDD4_K147R_ (Fig 1E, lane 3) and NEDD4_K535R_ (Fig 1E, lane 4) had no substantial effects on VP40 VLP production compared to WT NEDD4 (Fig 1E, lane 2) which indicated that acetylation of NEDD4 K667 may play a positive role in VP40 egress. In order to confirm this, we constructed a K-to-glutamine (Q) mutation of K667 (NEDD4_K667Q_), which mimics the acetylation state of NEDD4 and to test whether NEDD4_K667Q_ could enhance VP40 VLP production. E3 ubiquitin ligase activity of NEDD4 is indispensable for VP40 VLP production and cysteine (C)-to-alanine (A) mutation of C867 in NEDD4 (NEDD4_C867A_) results in abolishment of the E3 ubiquitin ligase activity of NEDD4, subsequently, leading to a dramatic decrease in VP40 VLPs [30], so we used NEDD4_C867A_ as a negative control. We transfected HEK293T cells with VP40 plus WT NEDD4, NEDD4_K667Q_ and NEDD4_C867A_ and found that NEDD4_K667Q_ could significantly increase VP40 VLP production (Fig 1F, compared lanes 2 and 3), where NEDD4_C867A_ could not (Fig 1F, compared lanes 2 and 4); this experiment further suggested that the acetylation of NEDD4 K667 could positively regulate VP40 VLP production.

Taken together, these results demonstrated the enhancement of cellular acetylation levels could facilitate the budding of EBOV VP40 VLPs, which required for NEDD4 K667 acetylation rather than VP40 acetylation.

### NEDD4 can be acetylated by P300

**Fig 2:**
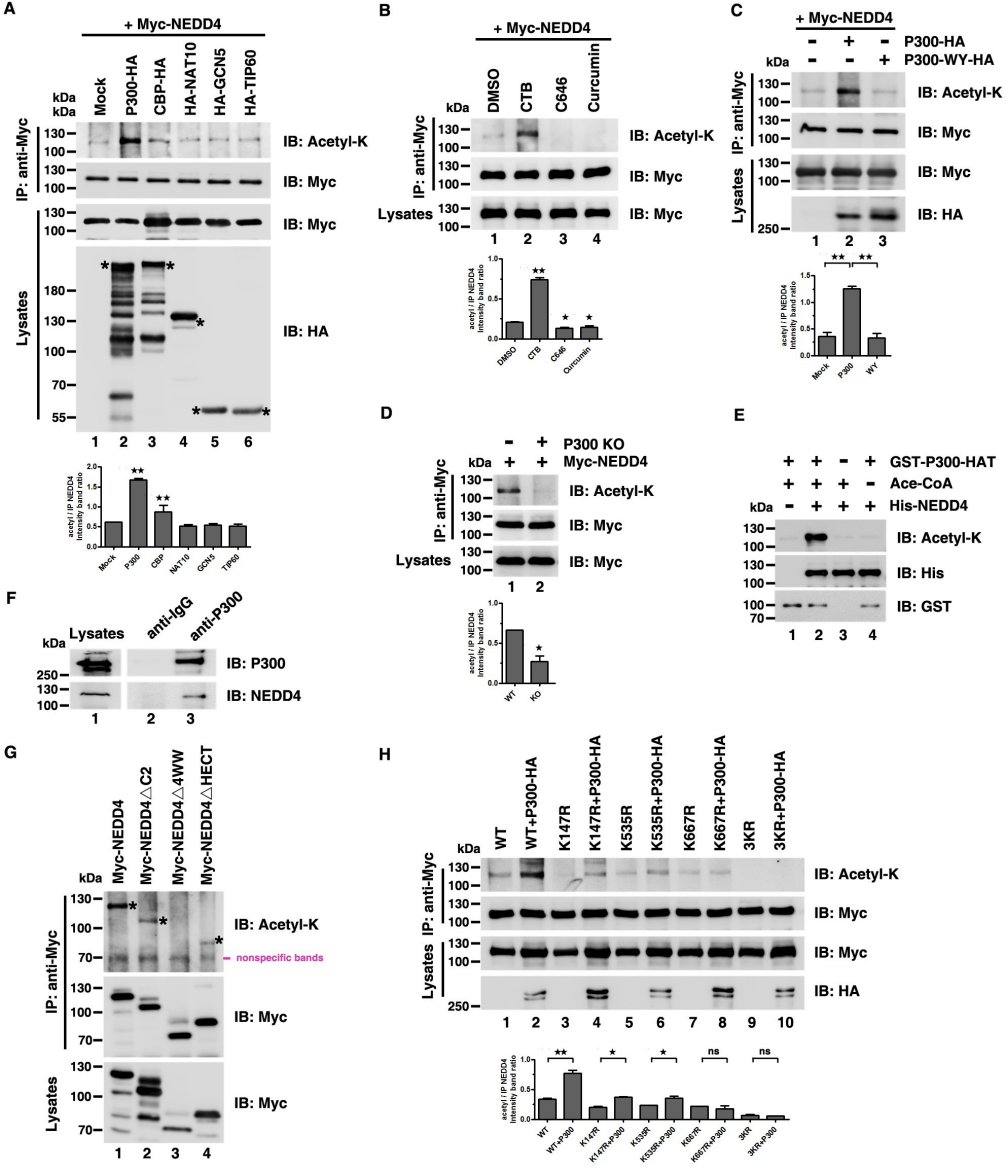
NEDD4 can be acetylated by P300. (A)-(C) NEDD4 acetylation in HEK293T cells was measured following overexpression of the indicated acetyltransferases targeted by “*” (A), treatment with the P300 activator CTB and inhibitors C646 and Curcumin (B), or overexpressing P300-HA and the acetyltransferase-inactive P300-WY-HA in P300 knockout (KO) cells (C). Cell lysates were immunoprecipitated with an anti-Myc antibody and analyzed via immunoblotting with an anti-Acetyl-K antibody to detect the acetylation of NEDD4. (D) NEDD4 acetylation was measured in HEK293T WT and P300 KO cells. (E) In vitro acetylation assays were measured by incubating purified His-NEDD4 with purified GST-P300-HAT followed by immunoblotting with an anti-Acetyl-K antibody to detect the acetylation of NEDD4. (F) Interaction between endogenous P300 and NEDD4. (G) Acetylation of Myc-NEDD4 and mutants was measured in HEK293T cells. (H) HEK293T cells were transfected with the indicated plasmid combinations to detect the influence of P300 on the acetylation of NEDD4 mutants. Error bars, mean ± SD of three experiments. Student’s t test; *p < 0.05; **p < 0.01; ***p < 0.001.

To further reveal the molecular mechanism of NEDD4 acetylation in EBOV VP40 egress, first, we aimed to identify the acetyltransferase responsible for NEDD4 acetylation. We ectopically expressed some classical acetyltransferases and found that, of these, P300 (Fig 2A, lane 2) could dramatically increase NEDD4 acetylation (Fig 2A). Besides, the specific P300 activator N-(4-chloro-3-tri-flfluoromethyl-phenyl)-2- ethoxy-benzamide (CTB) resulted in a dramatic increase in NEDD4 acetylation (Fig 2B, compared lanes 1 and 2) and conversely NEDD4 acetylation was abolished by using P300 inhibitor C646 or Curcumin (Fig 2B, lanes 3 and 4). These data indicated P300 may be an acetyltransferase of NEDD4. To further verify the specificity that P300 catalyzed NEDD4 acetylation, we transfected HEK293T cells with NEDD4 plus WT P300 or an acetyltransferase-dead P300 (P300-WY) lacking acetyltransferase activity [52]. We observed that overexpression of P300-WY could not increase NEDD4 acetylation (Fig 2C, compared lanes 2 and 3). Next, we used CRISPR/Cas9 to knockout (KO) endogenous P300 in HEK293T cells (S2A Fig) and found that NEDD4 acetylation was inhibited in P300 KO cells (Fig 2D). These results further suggested P300 was responsible for mediating NEDD4 acetylation in vivo.

To test whether P300 catalyzed for NEDD4 acetylation directly, we performed in vitro acetylation assays. In vitro system, we incubated purified recombinant His-NEDD4 with the P300 HAT domain (catalytic domain) purified from *Escherichia*. *coli* or with P300-HA that was immunoprecipitated from HEK293T cells. We found that His-NEDD4 could be acetylated by both P300 HAT (Fig 2E, compared lanes 2 and 3) and P300-HA (S2B Fig, compared lanes 1 and 2), but not the acetyltransferase-dead P300 (S2B Fig, compared lanes 1 and 3) or another purified recombinant acetyltransferase GST-PCAF (S2C Fig). These results demonstrated that P300 specifically acetylates NEDD4 in vitro.

The interaction between P300 and NEDD4 was critical for P300 catalyzing NEDD4 acetylation, so next we asked whether P300 interacted with NEDD4. We used co-immunoprecipitation assays to examine the NEDD4-P300 interaction, and found that ectopic NEDD4 could interact with ectopic P300 (S2D and S2E Fig); we also confirmed interaction between endogenous P300 and NEDD4 (Fig 2F). Next, to map the critical domain of NEDD4 that was necessary for its interaction with P300, we constructed three NEDD4 mutants (S2F Fig) and found that NEDD4△4WW failed to interact with P300 (S2G and S2I Fig). Then we asked whether NEDD4△4WW could be acetylated when NEDD4-P300 interaction was abolished. To do so, we transfected HEK293T cells with WT NEDD4 (Fig 2G, lane 1), NEDD4△C2 (Fig 2G, lane 2), NEDD4△4WW (Fig 2G, lane 3) and NEDD4△HECT (Fig 2G, lane 4) and found that only NEDD4△4WW could not be acetylated (Fig 2G), suggesting that NEDD4-P300 interaction was critical for P300-mediated NEDD4 acetylation. Next, we wanted to explore which acetylated site of NEDD4 was catalyzed by P300. In HEK293T cells, we co-expressed NEDD4 or its mutants in combination with P300 and found that P300 increased the acetylation of WT NEDD4, K147R and K535R, but not K667R and 3KR (Fig 2H). Together, these results provided evidence of P300 specifically catalyzing NEDD4 acetylation and responsible for the acetylation of NEDD4 K667.

### P300 regulates the release of VP40 VLPs

**Fig 3:**
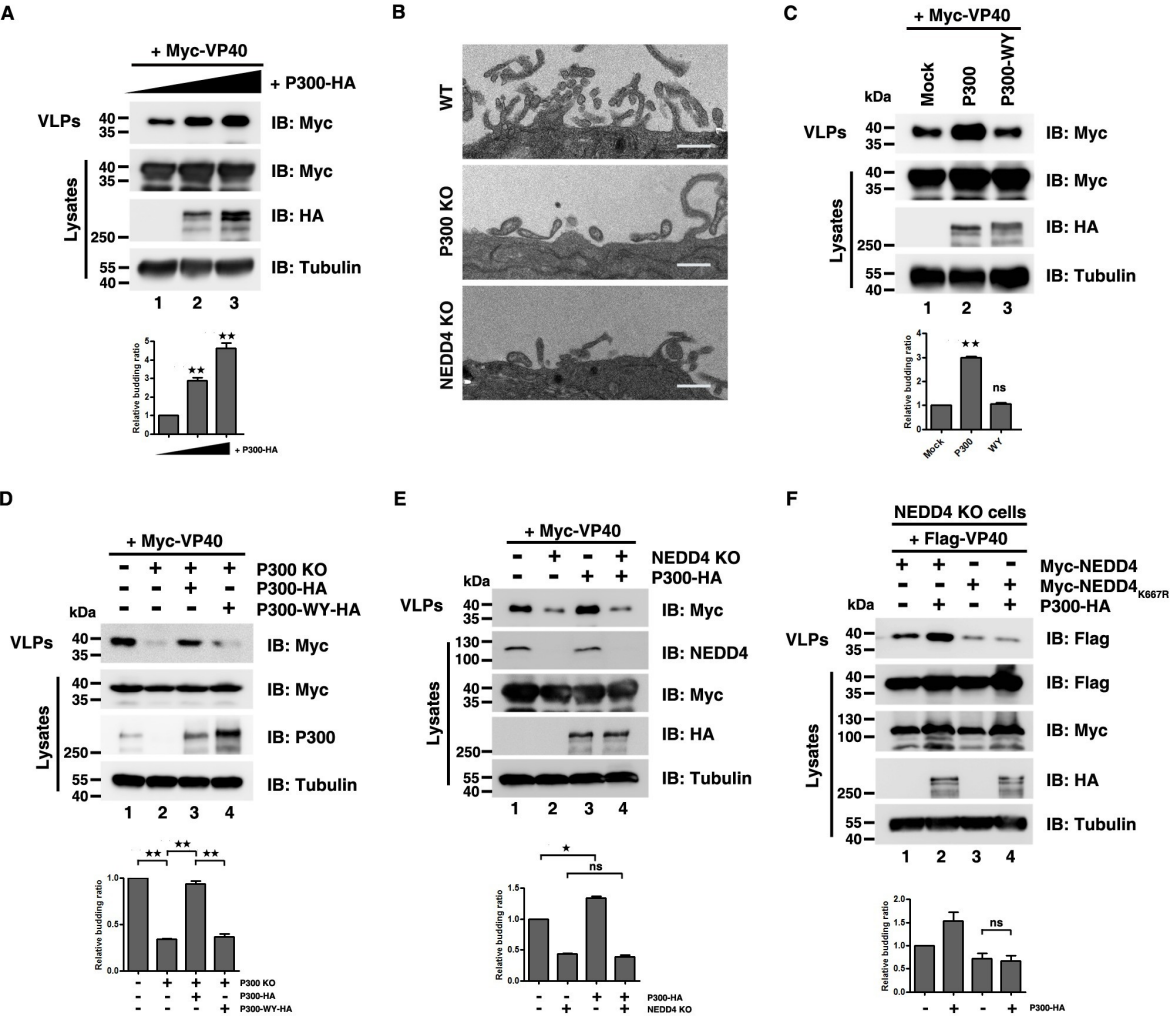
P300 regulates the release of VP40 VLPs. (A) HEK293T cells were transfected with increasing doses of P300-HA (0 μg, 1 μg and 3 μg) to detect its influence on VP40 egress. (B) The release of VP40 VLPs was measured using electron microscopy in HEK293T WT cells, P300 KO cells and NEDD4 KO cells. Scale bar, 500nm. (C)–(F) The release of VP40 VLPs was measured by overexpressing P300 acetyltransferase-dead mutant P300 WY-HA in P300 KO cell lines (C); by refreshing P300 and P300-WY levels in P300 KO cells (D); by overexpressing P300-HA in HEK293T NEDD4 KO cells (E); by overexpressing P300-HA, the NEDD4 WT and the NEDD4_K667R_ mutant in HEK293T NEDD4 KO cells (F). Error bars, mean ± SD of three experiments. Student’s t test; *p < 0.05; **p < 0.01; ***p < 0.001.

Since NEDD4 K667 was acetylated by P300 and the acetylation-mimicking mutant of NEDD4_K667Q_ promoted VP40 VLP production, we sought to explore whether enhancing NEDD4 acetylation by P300 could upregulate EBOV VP40 budding. First, we transfected VP40 plus some classical acetyltransferases in HEK293T cells to measure their influences on VP40 egress. We found that only P300 could enhance VP40 egress (S3A Fig). Furthermore, to determine whether the positive effects of P300 on VP40 VLP were dose-dependent, we transfected HEK293T cells with a constant amount of VP40 plus the increasing amounts of P300 and observed VP40 VLP production was increased with increasing doses of P300 (Fig 3A). Besides, the same and converse effects on VP40 egress was displayed by the P300 activator CTB (S3B Fig, lane 3) and inhibitors C646 and Curcumin (S3B Fig, lanes 4 and 5), respectively (S3B Fig). Next, we used transmission electron microscopy to visualize VP40 VLP egress directly and utilized CRISPR/Cas9 to knockout endogenous NEDD4 in HEK293T cells as a negative control (S3C Fig). The images showed that VP40 VLP egress was attenuated in P300 KO and NEDD4 KO cells in contrast to WT cells (Fig 3B). Additionally, the acetyltransferase-dead mutant P300-WY failed to promote VP40 budding (Fig 3C). Furthermore, in P300 KO HEK293T cells, VP40 VLP production was inhibited compared to WT HEK293T cells (Fig 3D, lanes 1 and 2) and exogenous expression of P300 (Fig 3D, lane 3), but not P300-WY (Fig 3D, lane 4), could rescue this inhibitory effect (Fig 3D). These data revealed that P300 could positively regulate the release of EBOV VP40 VLPs via its acetyltransferase activity.

The result showed that P300 specifically catalyzed for the acetylation of NEDD4 K667 (Fig 2H), so we sought to examine whether the P300-mediated VP40 egress was dependent on NEDD4 K667 acetylation. First, we observed that P300 lost the ability to promote VP40 VLP budding in the absence of NEDD4 (Fig 3E, compared lanes 3 and 4), indicating that NEDD4 was involved in the process of P300-mediated VP40 egress. Second, in NEDD4 KO cells, when NEDD4 and P300 were co-expressed, P300 significantly increased the VP40 VLP production (Fig 3F, compared lanes 1 and 2), but when co-expression of NEDD4_K667R_ and P300, P300 failed to increase the VP40 budding (Fig 3F, compared lanes 3 and 4), suggesting that P300-mediated regulation of the VP40 egress was dependent on NEDD4 K667 acetylation.

Taken together, these data proved a functional role for P300 as a positive regulator of VP40 VLP budding via catalyzing NEDD4 K667 acetylation.

### NEDD4 acetylation increases NEDD4-VP40 interaction

**Fig 4:**
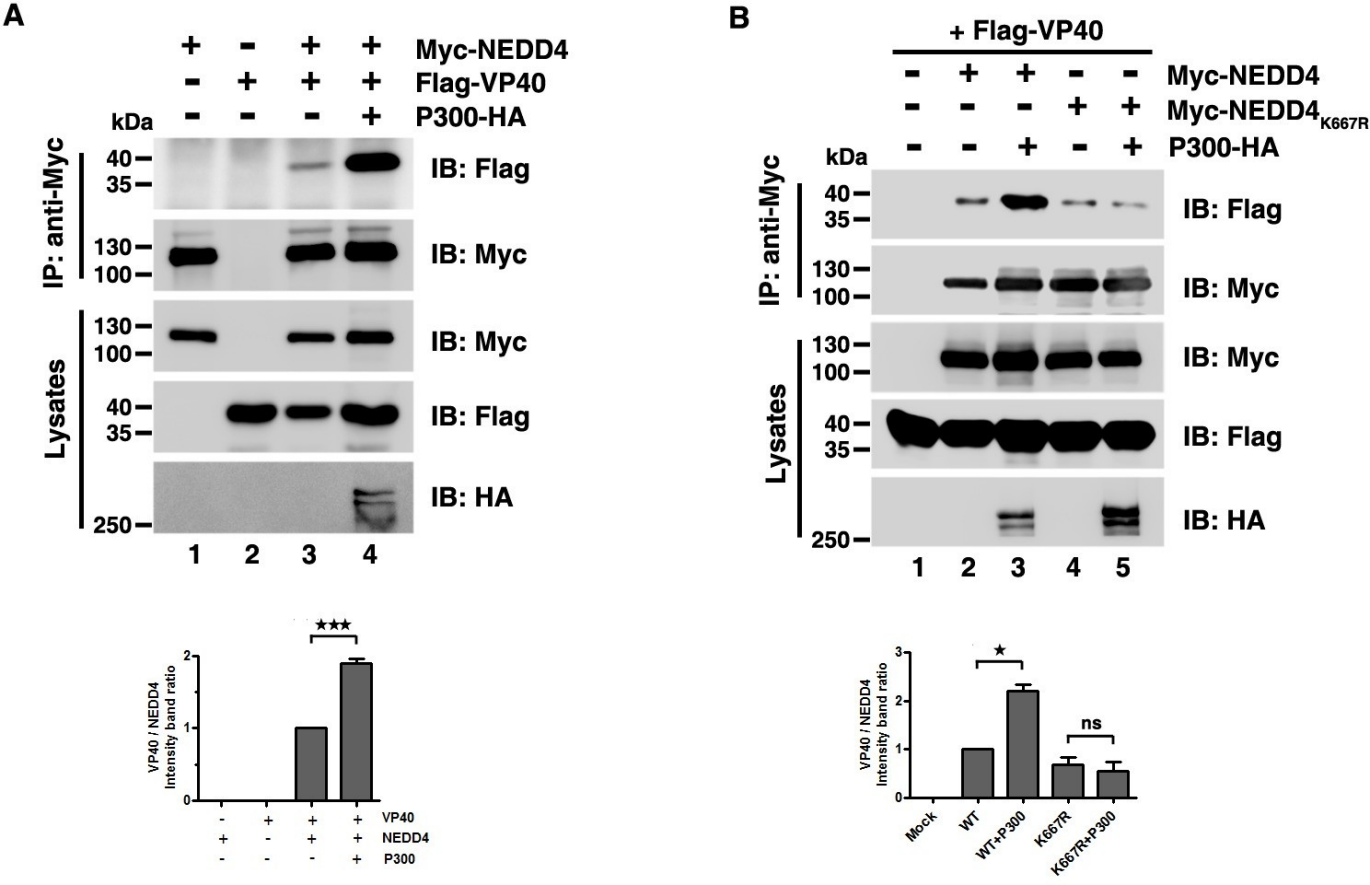
Acetylation enhances NEDD4-VP40 interactions. (A)-(B) Interactions were assayed by overexpressing the indicated plasmid combinations to determine the influence of P300 on VP40 -NEDD4 interaction or VP40- NEDD4_K667R_ interaction. Error bars, mean ± SD of three experiments. Student’s t test; *p < 0.05; **p < 0.01; ***p < 0.001.

We next asked how P300-mediated NEDD4 acetylation promoted VP40 VLP budding. Acetylation is known to have a critical effect in regulating protein-protein interactions [53], while NEDD4-VP40 interaction is indispensable for NEDD4-mediated VP40 VLP egress; thus, we hypothesized that P300-mediated NEDD4 K667 acetylation could result in increased NEDD4-VP40 interactions. Using an ecotopic expression system and co-immunoprecipitation assays, we found that NEDD4-VP40 interactions were increased when P300 co-expressed with NEDD4 and VP40 (Fig 4A, compared lanes 3 and 4). Besides, P300 had no effects on TSG101-VP40 interactions or BAG3-VP40 interactions (S4A and S4B Fig) which reflected the specificity in the enhancement of NEDD4-VP40 interactions by P300. Furthermore, NEDD4-VP40 interactions were impaired in P300 KO cells (S4C Fig). Additionally, we found that NEDD4_K667R_-VP40 interactions were weakened compared to the VP40-NEDD4 interactions (S4D Fig, compared lanes 2 and 3), and the ectopic expression of P300 failed to increase the VP40-NEDD4_K667R_ interactions (Fig 4B, compared lanes 4 and 5), suggesting that the enhancement of the NEDD4-VP40 interactions was mediated by P300-catalyzed NEDD4 K667 acetylation.

### NEDD4 acetylation enhances VP40 ubiquitination

Given that NEDD4 acetylation enhanced NEDD4-VP40 interactions, and NEDD4 is an E3 ligase that ubiquitinates VP40 and assists VP40 VLP budding, we hypothesized that the enhancement of NEDD4-VP40 interactions by acetylation could increase the ubiquitination of VP40, and subsequently enhance VP40 VLP egress.

**Fig 5:**
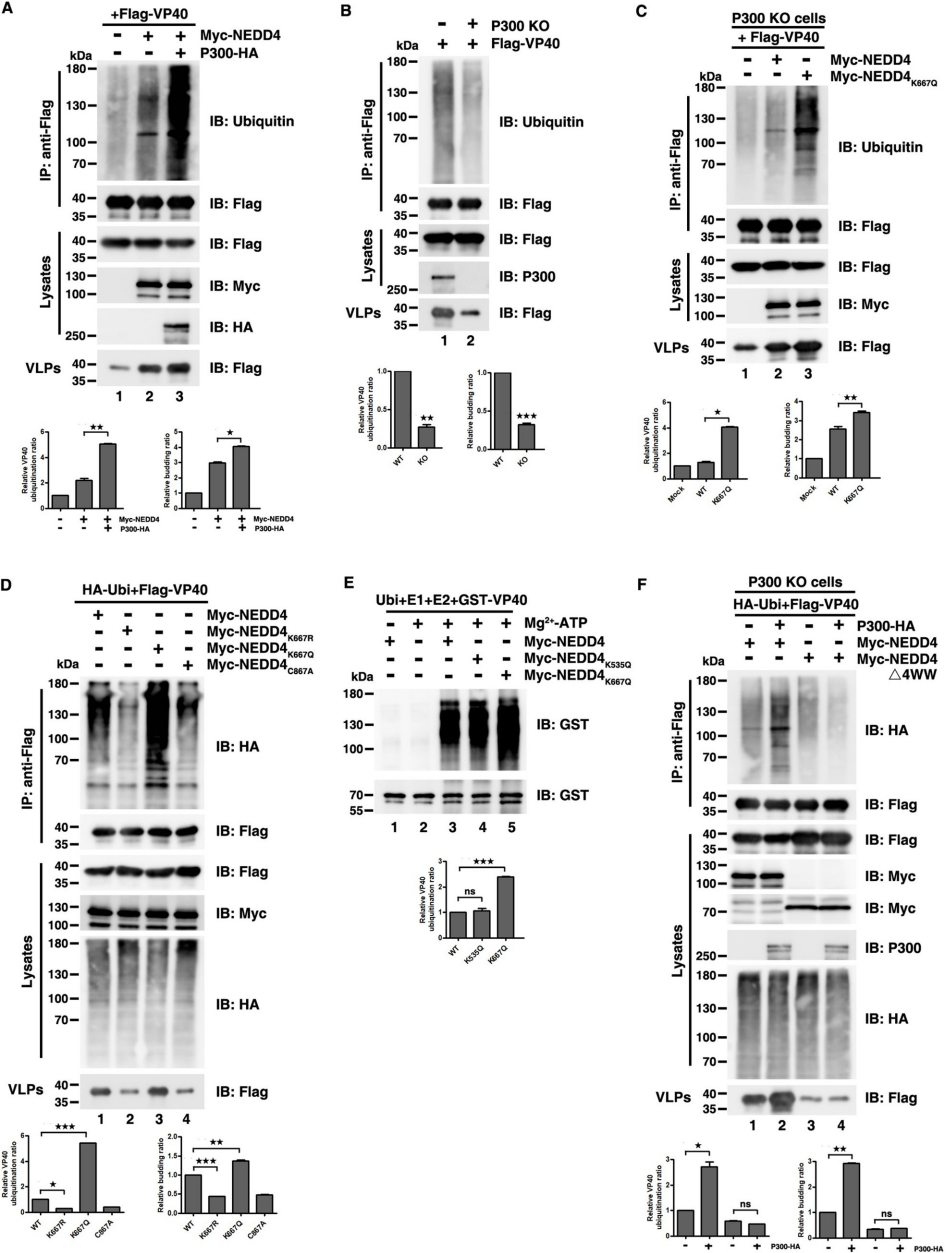
NEDD4 acetylation enhances VP40 ubiquitination. (A)-(C) Cells were transfected with the indicated plasmid combinations to measure endogenous ubiquitination of VP40 by overexpressing P300-HA in HEK293T cells (A), knocking out endogenous P300 (B), or overexpressing the NEDD4 WT and NEDD4_K667Q_ in P300 KO cells (C). (D) Cells were transfected with the indicated plasmid combinations to measure ubiquitination of VP40 by overexpressing the NEDD4 WT and mutants in HEK293T cells. (E) In vitro ubiquitination assays were used to measure purified GST-VP40 that was incubated with NEDD4 WT, NEDD4_K535Q_, and NEDD4_K667Q_, which were immunoprecipitated from HEK293T cells and then analyzed via immunoblotting with an anti-GST antibody to detect VP40 ubiquitination. (F) HEK293T P300 KO cells were transfected with the indicated plasmid combinations to detect the influence of P300 on the ubiquitination of VP40 when the interaction between NEDD4 and VP40 was removed. Error bars, mean ± SD of three experiments. Student’s t test; *p < 0.05; **p < 0.01; ***p < 0.001.

Indeed, we found that P300 dramatically increased VP40 ubiquitination levels which is consistent with the release of VP40 VLPs (Fig 5A, compared lanes 2 and 3; S5A Fig), while VP40 ubiquitination level was impaired in P300 KO cells (Fig 5B and S5B Fig) when using endogenously and exogenously expressed ubiquitin. Furthermore, the expression of acetylation-mimicking NEDD4_K667Q_ led to more intense VP40 ubiquitination compared to the expression of wild-type NEDD4 (Fig 5C, compared lanes 2 and 3; Fig 5D, compared lanes 1 and 3), while the expression of non-acetylatable NEDD4_K667R_ led to lower levels of VP40 ubiquitination compared to the expression of wild-type NEDD4 (Fig 5D, compared lanes 1 and 2). Besides, we conducted in vitro ubiquitination assays to confirm whether NEDD4 K667 acetylation promoted VP40 ubiquitination directly. We incubated recombinant GST-VP40 purified from *Escherichia*. *coli* with Myc-NEDD4, Myc-NEDD4_K535Q_ or Myc-NEDD4_K667Q_ which were immunoprecipitated from HEK293T cells. In Fig 5E, we observed that the VP40 ubiquitination levels were almost undetectable in the absence of Myc-NEDD4 (Fig 5E, lane 2); however, intense ubiquitination of VP40 was observed in the presence of Myc-NEDD4 (Fig 5E, lane 3) and the presence of Myc-NEDD4_K667Q_ (Fig 5E, lane 5) led to more intense ubiquitination of VP40 compared to Myc-NEDD4. Furthermore, the presence of another acetylation-mimicking mutant of NEDD4, Myc-NEDD4_K535Q_, which had no substantial effects on VP40 VLP production, only led to a similar degree of VP40 ubiquitination as that of Myc-NEDD4 (Fig 5E, compared lanes 3 and 4), suggesting that it was the P300-mediated acetylation of NEDD4 K667 that enhanced the levels of VP40 ubiquitination.

Previous study showed that the PPxY-type L domain of VP40 interacts with the WW domain of NEDD4 [9,26,30,35]. Thus, we asked whether NEDD4 acetylation failed to increase VP40 ubiquitination when NEDD4-VP40 interaction was abolished. To do so, in HEK293T P300 KO cells, we transfected VP40, P300 with WT NEDD4 or NEDD4△4WW which lacks interactions with VP40 and found that P300 could increase VP40 ubiquitination with WT NEDD4 (Fig 5F, compared lanes 1 and 2), but failed to increase VP40 ubiquitination with NEDD4△4WW (Fig 5F, compared lanes 3 and 4). The same effect could be observed when L domain of VP40 was lacked (S5C Fig, compared lanes 3 and 4).

In summary, these findings demonstrated that ubiquitination of VP40 could be strengthened by P300-mediated NEDD4 K667 acetylation which was required for NEDD4-VP40 interaction.

### NEDD4 acetylation alters VP40 subcellular localization

**Fig 6:**
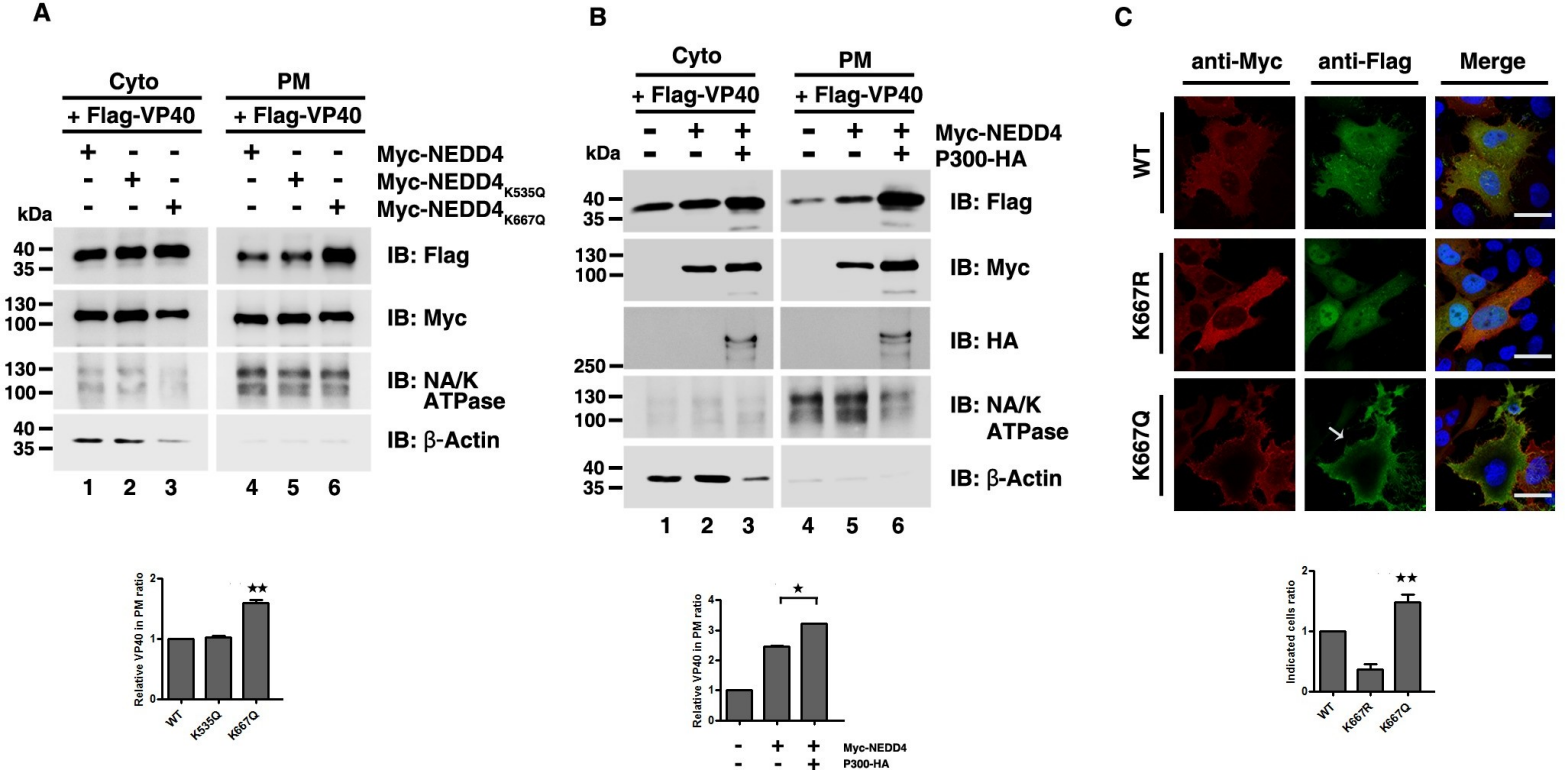
NEDD4 acetylation alters VP40 subcellular localization. (A) Separation of the cytoplasm and the plasma membrane was conducted to detect the influence of the acetylation-mimicking mutants of NEDD4 on the plasma membrane localization of VP40. (B) Separation of the cytoplasm and the plasma membrane was conducted to detect the influence of P300 on the plasma membrane localization of VP40. (C) HeLa cells were transfected with the indicated plasmid combinations to visualize VP40 localization. The white arrow represents the localization pattern of VP40 at the plasma membrane and in PM projections. DAPI (blue) was used to stain nuclear DNA. Scale bar, 10 μm. Error bars, mean ± SD of three experiments. Student’s t test; *p < 0.05; **p < 0.01; ***p < 0.001.

Efficient egress of VLPs requires the localization of VP40 at the plasma membrane (PM) [54] and ubiquitination can be regarded as a signal to drive VP40 migration to the PM [33]. Since NEDD4 acetylation enhanced VP40 ubiquitination, we speculated acetylation-mediated enhancement of VP40 ubiquitination could change VP40 localization and drive a larger VP40 migration to the PM. In order to verify our guess, first, we used a biochemical approach to determine the levels of VP40 in PM fractions. HEK293T cells were transfected with VP40 plus WT NEDD4, NEDD4_K535Q_ and NEDD4_K667Q_ and both cytosol and PM fractions were separated and subjected to western blot analysis. In Fig 6A, we found that the amount of VP40 in the PM was increased in the presence of NEDD4_K667Q_ (Fig 6A, lane 6) compared to WT NEDD4 (Fig 6A, lane 4), but not in the presence of NEDD4_K535Q_ (Fig 6A, compared lanes 4 and 5). NA/K ATPase was used as a PM control and β-actin was used as a cytosol control (Fig 6A). Besides, when we co-expressed P300 and NEDD4, we observed that the amount of VP40 in PM was increased compared to the single expression of NEDD4 (Fig 6B, compared lanes 5 and 6), which further suggested that an acetylation-mediated increase in VP40 ubiquitination led to increased VP40 in the PM. Next, we utilized confocal microscopy to visualize VP40 localization in the presence of NEDD4, NEDD4_K667R_ and NEDD4_K667Q_; the images showed that more VP40 was distributed around the cell periphery and to the PM projections in the presence of NEDD4_K667Q_ compared to in the presence of NEDD4 or NEDD4_K667R_ (Fig 6C). These results suggested that the accumulation and localization of EBOV VP40 in the PM projections could be increased by NEDD4 acetylation and subsequently resulted in enhanced egress of VP40 VLPs.

### Zaire ebolavirus production is lessened in P300 KO cells

**Fig 7:**
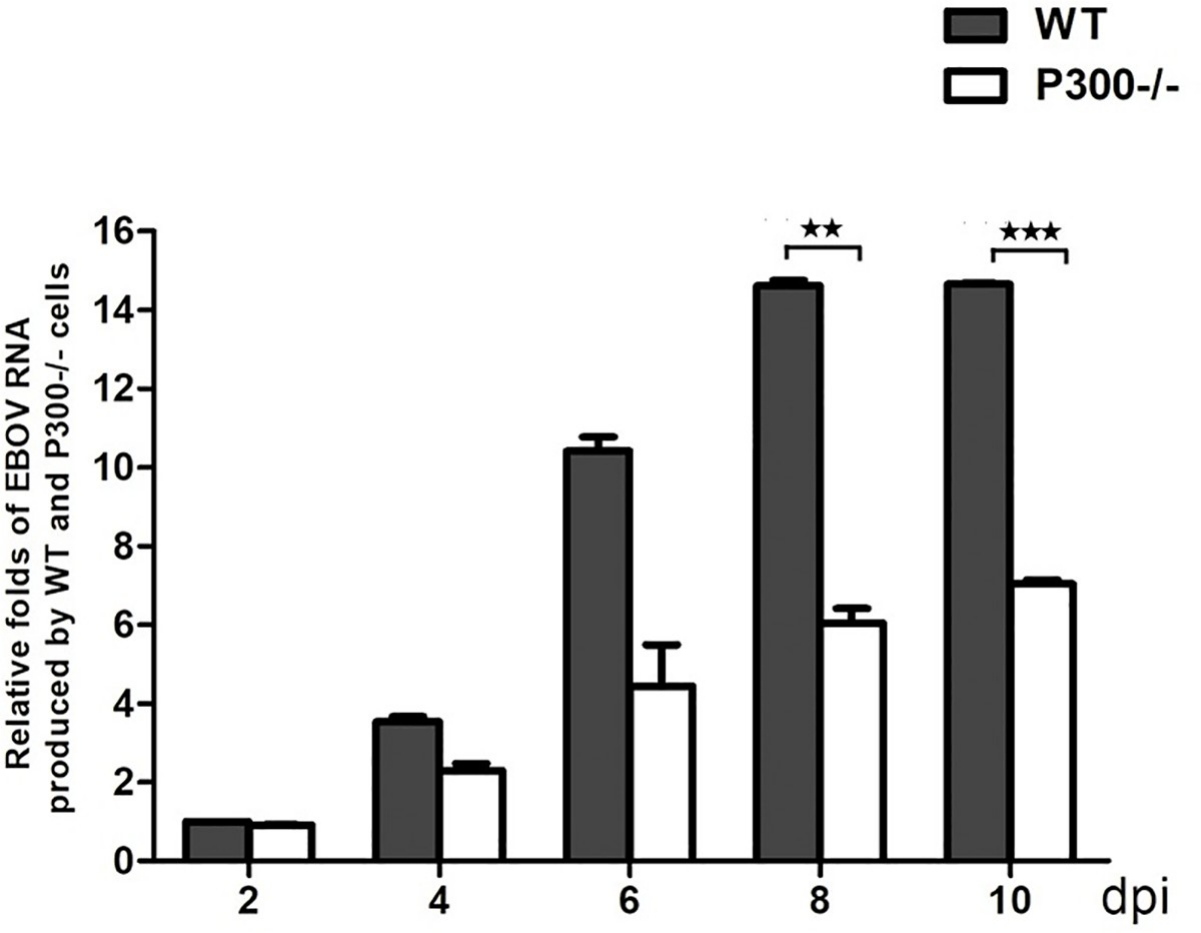
Zaire ebolavirus production is lessened in P300 KO cells. HEK293T WT cells and P300 KO cells were infected with the Zaire ebolavirus at a multiplicity of infection (MOI) of 1. Aliquots of supernatant fluids were taken at days 2, 4, 6, 8 and 10 for the viral RNA qPCR determination.

Having found that P300-mediated NEDD4 acetylation enhanced the VP40 VLP production, we next sought to determine whether NEDD4 acetylation was required for the productive replication of Zaire ebolavirus. EBOV is a biosafety level 4 (BSL4)virus and doing relevant experiments needs to be in the BSL4 laboratory. To do so, HEK293T wild type cells and P300 knockout cells were infected by Zaire ebolavirus at an MOI of 1 in Wuhan National Laboratory for Biosafety and then aliquots of supernatant fluids were taken at days 2, 4, 6, 8 and 10 after infection for viral RNA qPCR determination. As a result, we found Zaire ebolavirus production in WT cells was more than 8 folds as big as that in P300 KO cells at 8 and 10 days after infection (Fig 7), suggesting that P300 was critical for the productive replication of Zaire ebolavirus and may have a physiological impact on the EBOV life cycle.

## Discussion

**Fig 8:**
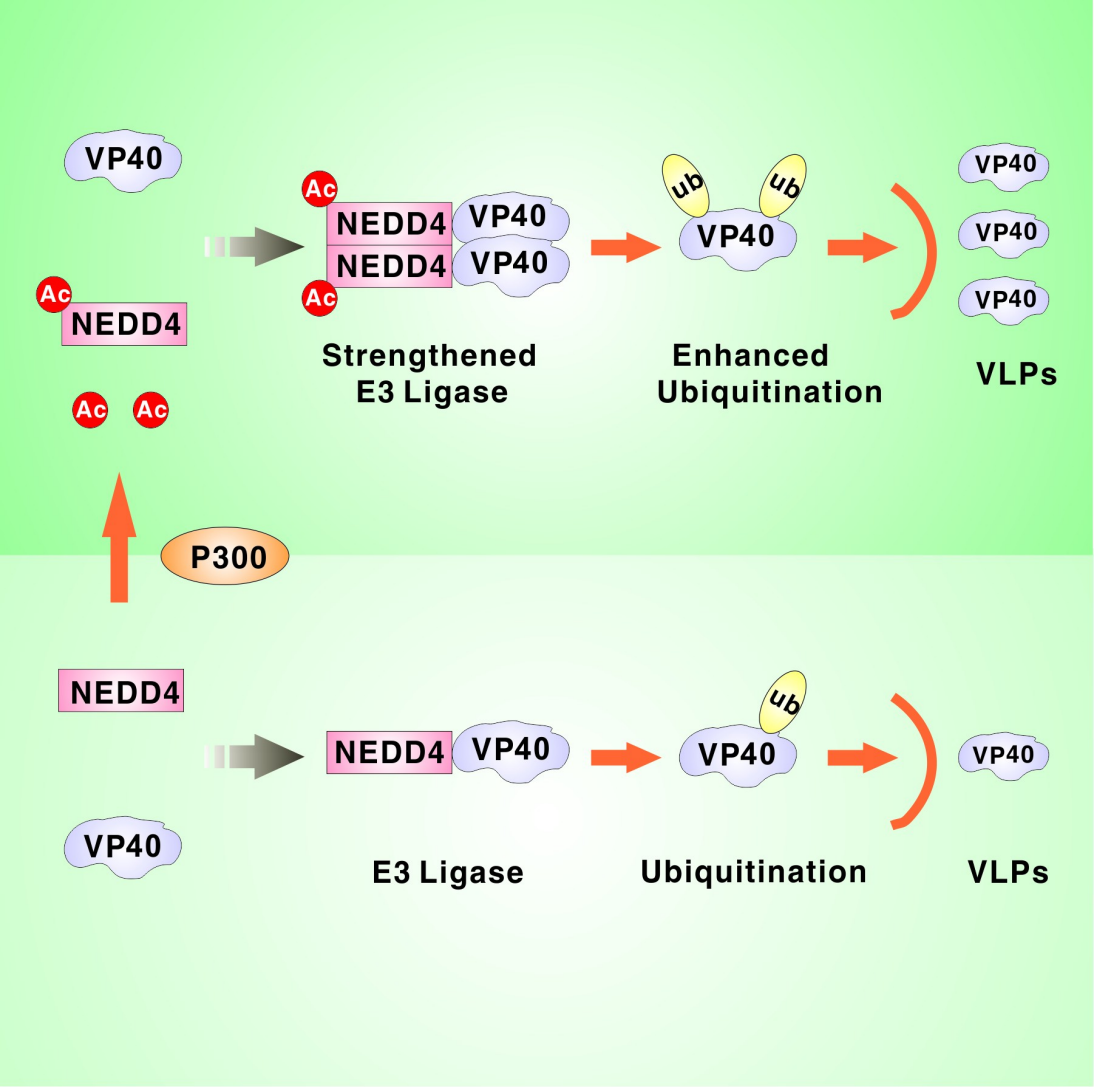
Model of the NEDD4 acetylation that promoted the release of VP40 VLPs.

A substantial amount of literature has been generated that advances our understanding of the relationship between acetylation and viral infection. Post-translational modifications have been reported to regulate EBOV VP40 egress, especially ubiquitination, but acetylation has not been investigated yet. In our study, we found that the enhanced cellular acetylation by HDAC deacetylase inhibitors promoted EBOV VP40 egress (Fig 1A and S1A Fig) and we examined the formation of acetylated VP40 via immunoprecipitation assays (S1B Fig); however, the mutation results showed that the acetylation of VP40 did not influence the release of VP40 VLPs (S1C Fig). Therefore, we hypothesized that the acetylation of host cellular factors might participate in the release of VP40 VLPs. We screened some cellular factors that interact with VP40 and found that NEDD4 acetylation positively regulated VP40 egress. We identified three sites of lysine acetylation on NEDD4 (Fig 1C) and found that an acetylation-mimicking mutation of NEDD4 K667 facilitated EBOV VP40 budding (Fig 1E and 1F). Furthermore, we revealed that acetyltransferase P300 was responsible for acetylating NEDD4 at K667 which enhanced VP40 egress (Figs 2 and 3A). When NEDD4_K667R_ was expressed in NEDD4 KO cells, P300 failed to increase VP40 budding (Fig 3F). These results demonstrated that P300-mediated regulation of EBOV egress was dependent on NEDD4 K667 acetylation. In the context of the molecular mechanism by which NEDD4 acetylation promoted the release of EBOV VLPs, we found that NEDD4 acetylation could strengthen NEDD4-VP40 interactions (Fig 4) and the enhancement of NEDD4-VP40 interactions by acetylation could increase the ubiquitination of VP40 (Fig 5), and subsequently enhance VP40 VLP egress. Finally, we found that Zaire ebolavirus productive replication was dramatically reduced in P300 knockout cell lines, suggesting that NEDD4 acetylation may have a physiological effect on Ebola virus life cycle (Fig 7). The results of this study have significant implications regarding a general mechanism by which P300-mediated NEDD4 acetylation is involved in the NEDD4-VP40 interaction, VP40 ubiquitination and egress (Fig 8).

Multiple interactions between viruses and the acetylation machinery result in the direct targeting of viral proteins for acetylation modifications, as observed in, e.g., human papillomavirus (HPV), HIV-1 and IVA [55–57]. The acetylation of viral proteins assists viruses by dampening antiviral responses and enhancing viral replications. In our study, we found that EBOV VP40 was acetylated in cells at K221, K224, K225, K274 and K275 [49]. These sites are located on a basic surface of the VP40 CTD, which is important for interacting with the negatively charged cytoplasmic leaflet of the cell membrane [14]. We found that K- to -R mutants of VP40 at different lysines budded at the same levels as wild-type (S1C Fig), which indicated that the significance of VP40 acetylation was not in the budding process. Notably, this phenomenon could be explained by recognizing that the K- to -R mutation of VP40 conserved its positive charge so that the mutants could still trigger electrostatic interactions with the cellular membrane. A previous study revealed that VLP budding was abolished in an Ebola VP40 K274E/K275E mutant in which the positively charged lysines had been replaced with negatively charged residues, which further supports our results [58]. We believe that acetylation influenced EBOV VP40 VLPs in a manner that was dependent on the NEDD4-induced signaling process rather than VP40 acetylation itself.

P300 has emerged as a potential therapeutic target for viral infection and cancer [59]. Nevertheless, it is a challenging drug target since substrate-binding site of P300 and most inhibitory compounds characterized to date have been found to target the acetyl-CoA binding site in the enzyme [60]. The P300 phytochemical inhibitor curcumin, which has multiple functions such as antioxidant and anti-inflammatory functions [61], has also been found to have antiviral effects. For example, curcumin inhibits Zika and chikungunya virus infection by inhibiting cell binding [62]. Furthermore, curcumin can inhibit IAV in vitro and alleviate the severity of disease in mice after IAV infection [63,64]. Importantly, curcumin has been thought to cure patients infected with EBOV through its suppression of cytokine release and the cytokine storm [65]. In our study, we found that curcumin could negatively regulate NEDD4 acetylation and EBOV VP40 VLPs (Fig 2B and S3B Fig). These results revealed inhibitory effect of curcumin on EBOV productive replication, which further implied that curcumin could be regarded as an attractive candidate drug for the treatment of Ebola virus disease.

NEDD4 K667 acetylation could enhance interactions with VP40, which could only be explained by acetylation-induced conformational changes in NEDD4 that allowed better accessibility for interacting with VP40. Currently, only the structure of NEDD4 HECT domain is resolved, thus, we were unable to directly show the concrete mechanism by analyzing the structure of NEDD4. In attempt to answer the question, we used more complete structural data of NEDD4-like E3 ligase WWP2 to explain how NEDD4 K667 acetylations affected interactions with VP40. By analyzing the structure of WWP2 -WW2-2,3-linker-HECT (PDB ID: 5TJ7) [66], we found that the HECT domain of WWP2 is mainly composed of α-helixes and WWP2 WW2 domain is closed to the HECT domain. WWP2 K637 is conserved with NEDD4 K667 and locates in the centre of HECT domain. In addition, WWP2 K637 is a basic amino acid and can provide positive charge to promote the formation of a salt bridge and stabilize the whole conformation of HECT. When WWP2 K637 was mutated to electrically neutral Q, the salt bridge was broken. Therefore, the conformation of WWP2 HECT was more flexible, which made the following WW2 domain patulous and swaying. Conformation changes would present different solvent accessible area and different topological contacts with substrate proteins. According to the proposed model of WWP2, we speculated that the enhanced VP40 interactions with NEDD4 could be ascribed to the fact that NEDD4 K667 acetylation changed the conformation of its HECT domain, leading to the NEDD4 4WW domains more flexible to associate with VP40.

Accumulating evidence has demonstrated that protein acetylation and ubiquitination can modulate each other through different mechanisms, such as through direct competition for lysine binding or via protein interactions [67–69]. In our study, we discover that a functionally characterized acetylation site of NEDD4 is responsible for enhancing VP40 ubiquitination and contributing to VP40 egress. This observation is the first demonstration that PTM crosstalk can integrate diverse signals and vastly increase their regulatory potential in the EBOV life cycle. Furthermore, our findings provide insights into understanding the epigenetic molecular mechanisms that regulate viral egress through post-translational modifications.

## Materials and methods

### Cell lines

HEK293T, HEK293T P300 KO cells, HEK293T NEDD4 KO cells and HeLa cells were cultured in Dulbecco’s modified Eagle’s medium (DMEM, Gibco) with 10% fetal bovine serum (FBS, Gibco). All cells were cultured at 37°C in a humidified incubator with 5% CO_2_.

### Antibodies and reagents

Rabbit monoclonal anti-NEDD4 (cat.no.5344), rabbit monoclonal anti-P300 (cat.no.86377), rabbit polyclonal anti-Acetylated-Lysine (cat.no.9441), mouse monoclonal anti-GST (cat.no.2624) and mouse monoclonal anti-His (cat.no.12698) were purchased from Cell Signalling Technology. Rabbit polyclonal anti-Ubiquitin (cat.no.110433), rabbit polyclonal anti-NA/K ATPase (cat.no.A12405), and mouse monoclonal anti-β-Actin (cat.no.AC004) were purchased from ABclonal. Mouse monoclonal anti-HA (cat.no.M180) and mouse monoclonal anti-Myc (cat.no.M192) were purchased from MBL. Mouse monoclonal anti-Flag (cat.no.F1804) and mouse monoclonal anti-Tubulin (cat.no.T6199) were purchased from SIGMA. Goat anti-rabbit IgG HRP conjugated (cat.no.31460), goat anti-mouse IgG HRP conjugated (cat.no.31430), goat anti-rabbit IgG Rhodamine (cat.no.31670) and goat anti-mouse IgG Fluorescein (cat.no.31569) were purchased from Invitrogen.

After transfected with indicated plasmids, HEK293T cells were treated by TSA (1 μM), SAHA (3 μM) and NAM (10 mM) for 8–10 hours before collecting supernatants for ultracentrifugation of the VLP assay. Cells were pretreated by CTB (50 μM), C646 (10 μM), and Curcumin (10 μM) for 6 hours and then transfected with indicated plasmids to conduct VLP assay or to detect NEDD4 acetylation. The nucleic acid stains (Super GelRed, NO.:S-2001) were purchased from US Everbright Inc (Suzhou, China). The Cell Counting Kit (cat.no.ZP328) was purchased from ZOMANBIO (Beijing, China).

### Knockout cell lines construction

HEK293T P300 KO cells or NEDD4 KO cells were constructed by transient transfection of PX459-P300-KO or PX459-NEDD4-KO plasmid followed by selection with 1 μg/ml puromycin. The surviving cells were sorted out as the monoclone after 5 days later. Human P300-targeted sgRNA: TAGTTCCCCTAACCTCAATA; Human NEDD4-targeted sgRNA: TACTGGGGCCTCCGACTCGT.

### Immunoblotting and immunoprecipitation

Cell lysates were collected in lysis buffer (150 nM NaCl, 50 nM Tris-HCl pH 7.4, 1% Triton X-100, 1mM EDTA pH 8.0, 0.1% SDS) supplemented with protease inhibitor cocktail. Proteins in cell lysates were separated by SDS-PAGE and analyzed by immunoblotting.

For immunoprecipitation, cells were lysed by lysis buffer containing protease inhibitor cocktail for 30 min on ice and then centrifuged at 13,000 rpm for 30 minutes at 4°C. The supernatants were immunoprecipitated by the indicated antibody for 12 h at 4°C. The immunoprecipitants were washed by lysis buffer four times and then boiled in 1x SDS-loading buffer for immunoblotting. To detect VP40 and NEDD4 acetylation, lysis buffer was added 1 μM TSA and 10 mM NAM.

### Virus-like particle assay

HEK293T cells were transfected with indicated plasmids and then culture mediums were collected and centrifuged at 13,000 rpm for 5 minutes to remove cell debris. The centrifuged supernatants were layered onto 20% (w/v) sucrose and ultracentrifuged at 35,000 rpm for 2 h at 4°C. The VLP pellets were resuspended in PBS overnight at 4°C and were analyzed by immunoblotting.

### Recombinant protein purification

*Escherichia coli* strain, BL21, was transformed with the indicated plasmid encoding GST-VP40. And then the monoclonal cell was first grown overnight in LB medium supplemented with 50 μg/mL ampicillin at 37°C. 5 mL overnight cultured bacteria were then inoculated into 100 mL LB medium for further culture. When the OD600 reached 0.6, protein expression was induced at 16–18°C overnight by adding 0.5 mM IPTG. Cells were harvested after induction, and were sonicated in PBS buffer. The lysates were centrifuged at 8000 rpm for 15 minutes at 4°C. After centrifugation, GST-VP40 proteins were purified with glutathione resins (GenScript, L00206) according to the manufacture’s protocols.

### In vitro acetylation and ubiquitination assay

For vitro acetylation assay, purified His-NEDD4 (5 μg) was incubated with purified GST-P300-HAT (1 μg), purified GST-PCAF (1 μg) or P300-HA and P300-WY-HA immunoprecipitated from cell lysates with adding acetyl-coenzyme A (20 μM) and 5 x HAT assay buffer [250 mM Tris–HCl pH 8.0, 50% (v/v) glycerol, 0.5 mM EDTA, 5 mM dithiothreitol] in a total volume of 50 μL. The mixture was mixed mildly and placed for 2 h at 30°C. Then the contents were boiled in 1x SDS-loading buffer for immunoblotting with anti-acetyl-lysine antibody to detect NEDD4 acetylation.

For vitro ubiquitination assay, purified GST-VP40 was incubated with Myc-NEDD4, Myc-NEDD4_K535Q_ and Myc-NEDD4_K667Q_ immunoprecipitated from cell lysates in the presence of purified ubiquitin, E1, E2, Mg^2+^-ATP and DTT (50 mM) in a total reaction volume of 50 μL. The contents were mixed gently and incubated at 37°C for 1 h. Quench assays by addition of 50μL 2x SDS loading buffer followed by heating to 95°C for 5 minutes or 70°C for 10 minutes. The ubiquitination of VP40 was analyzed by immunoblotting with anti-GST antibody.

### Immunofluorescence

Cells were fixed with 4% (w/v) paraformaldehyde for 20 minutes at room temperature and were washed by PBS for three times. Then cells were permeated by 0.1% (v/v) Triton X-100 for 20 minutes at room temperature and were washed by PBS for three times. Then cells were blocked by 3% (v/v) BSA for 30 minutes. After blocking, cells were incubated with indicated primary antibodies for 1 h and then washed by 1% (v/v) BSA for three times. The second fluorescent antibodies were added for another 1 h. DAPI was used to stain nucleus for 5 minutes.

### Transmission electron microscopy

HEK293T wild type and knockout cells were transfected with VP40 for 48 hours and then were fixed by fixative liquid [3% (v/v) paraformaldehyde, 1.5% (v/v) glutaraldehyde, 2.5% (w/v) sucrose in 0.1M sodium phosphate buffer, pH 7.4] for 1 h at room temperature. Then the cells were collected and subjected to gradient centrifugation (1000 g, 5 min; 3000 g, 5 min; 6000 g, 5 min; 12000 g, 5 min) at 4°C. Post-fixed with 1% osmium tetroxide for 1 h on ice under dark conditions, cells were incubated with 2% uranyl acetate overnight, dehydrated in increasing concentrations of ethanol (50%, 75%, 95%, and 100%) and processed for embedding in epoxy resin. Ultrathin (70 nm) sections were collected on uncoated 200-mesh copper grids and stained with uranyl acetate and lead citrate, and were observed by transmission electron microscopy (JEOL, JEM-1400 plus) operating at 100kV.

### Infectivity assay and analysis by quantitative polymerase chain reaction

HEK293T wild type cells and P300 knockout cells were infected by Zaire ebolavirus for 2 hours at an MOI of 1 and then the medium was replaced with fresh DMEM. Aliquots of supernatant fluids were taken at days 2, 4, 6, 8 and 10 after infection for viral RNA qPCR determination.

For qPCR assay, the viral RNAs in supernatants were extracted by viral RNA mini kit (QIAamp, cat.no.52906). Then 0.5 μg of viral RNA was used for reverse transcription (thermoscientific, cat. no. K1682) and analyzed by qPCR (ABclonal, cat. no. RK21203) to measure Zaire Ebola virus RNA abundance. The following primers were used: Zaire Ebola virus VP40 forward: 5’-ACCAGGCAGTGTGTCATCAG-3’; Zaire Ebola virus VP40 reverse: 5’-TTGGTTGCCTTGCCGAAATG-3’.

### Subcellular fractionation

After transfected indicated plasmids, cell culture medium was discarded and cells were washed by PBS containing CaCl_2_ and MgCl_2_ pH 8.0 for three times. Cells were then placed for 30 minutes at 4°C with adding 0.25 mg/mL biotin solution. After incubation, cells were washed by PBS containing 1 M glycine for quenching uncombined biotin. Then, cells were collected and lysed by lysis buffer for 30 minutes at 4°C and subsequent centrifuged at 13,000 rpm for 30 minutes at 4°C. The supernatants were incubated with chained penicillin agarose beads for rotating overnight at 4°C for immunoprecipitated biotin-combined proteins. The beads were then washed by PBS for five times and were boiled by addition of 30 μL 2x SDS loading buffer for 10 minutes. The results were analyzed by immunoblotting.

### Mass spectrum

NEDD4 acetylation sites were detected by transient expression of NEDD4 in HEK293T cells. HEK293T cells were transfected with Myc-NEDD4 treating 1 μM TSA. 36 h post transfection, cells were washed and lysed by lysis buffer including 1 μM TSA and 10 mM NAM. Following sonication and centrifugation, the supernatants were immunoprecipitated by Myc antibody for 12 h at 4°C. The immunoprecipitants were washed by lysis buffer four times and then subjected to SDS–PAGE. Corresponding gel bands of acetylated NEDD4 were excised and digested with trypsin. The digested peptides were analyzed by mass spectrometry.

### Statistical analysis

Statistical parameters including the definition and exact values of n, distribution and deviation are reported in the Figure legends. Data are expressed as mean ± standard deviation (SD). The significance of the variability between different groups was determined by two-tailed unpaired Student’s t test of two groups using GraphPad Prism software (version 5.0). A p value of < 0.05 was considered statistically significant and a p value of > 0.05 was considered statistically non-significant.

## Supporting information

S1 Fig (A) The release of VP40 VLPs was measured after adding TSA. (B) HEK293T cells were transfected with vectors or Myc-VP40 immunoprecipitated with an anti-Acetyl-K antibody and analyzed via immunoblotting with an anti-Myc antibody to detect the acetylation of VP40. (C) Measurement of the release of VP40 and mutant VLPs. (D) The conserved sites are K535 and K667. Error bars, mean ± SD of three experiments. Student’s t test; *p < 0.05; **p < 0.01; ***p < 0.001.
(TIF)

S2 Fig (A) HEK293T P300 knockout cells were analyzed using immunoblotting. (B) In vitro acetylation assays were used to measure purified His-NEDD4 that was incubated with P300-HA and P300-WY-HA, which were immunoprecipitated from HEK293T cells and then analyzed using immunoblotting with an anti-Acetyl-K antibody for detecting the acetylation of NEDD4. (C) An in vitro acetylation assay was used to measure purified His-NEDD4 that was incubated with purified GST-PCAF followed by detection of NEDD4 acetylation. (D) and (E) Interactions between P300-HA and Myc-NED4. The specific band of P300 is indicated by a “*”. (F) Schematic drawing of the NEDD4 WT and mutants. (G)-(I) Interactions between the P300-HA and Myc-NED4 mutants. The specific band of P300 is indicated by a “*”.
(TIF)

S3 Fig (A) The release of VP40 VLPs was measured when overexpressing acetyltransferases. The target bands of acetyltransferases are indicated by a “*”. (B) The release of VP40 VLPs was measured in response to treatment with P300 activators and inhibitors in the HEK293T WT cell lines. (C) HEK293T NEDD4 knockout cells were analyzed using immunoblotting. Error bars, mean ± SD of three experiments. Student’s t test; *p < 0.05; **p < 0.01; ***p < 0.001.
(TIF)

S4 Fig (A)-(B) Interactions were measured by overexpressing the indicated plasmid combinations to detect the influence of P300 on the interactions between VP40 and TSG101(A) or BAG3(B). (C) Interaction between VP40 and NEDD4 in HEK293T P300 KO cell lines.(D) Interaction between VP40 and NEDD4 or NEDD4_K667R_ in HEK293T WT cell lines.
(TIF)

S5 Fig (A)-(C) Cells were transfected with the indicated plasmid combinations to measure the exogenous ubiquitination of VP40 by overexpressing P300-HA and HA-ubiquitin in HEK293T cells (A), Myc-NEDD4 and HA-ubiquitin in P300 KO cells (B), or overexpressing P300-HA when the interaction between VP40 and NEDD4 was impaired in P300 KO cells (C). Error bars, mean ± SD of three experiments. Student’s t test; *p < 0.05; **p < 0.01; ***p < 0.001.
(TIF)
