# Supplementary figures and images for: P300-mediated NEDD4 acetylation drives ebolavirus VP40 egress by enhancing NEDD4 ligase activity
Source: PLoS Pathog. 2021 Jun 10;17(6):e1009616. doi: 10.1371/journal.ppat.1009616 (PMC8191996; doi:10.1371/journal.ppat.1009616)

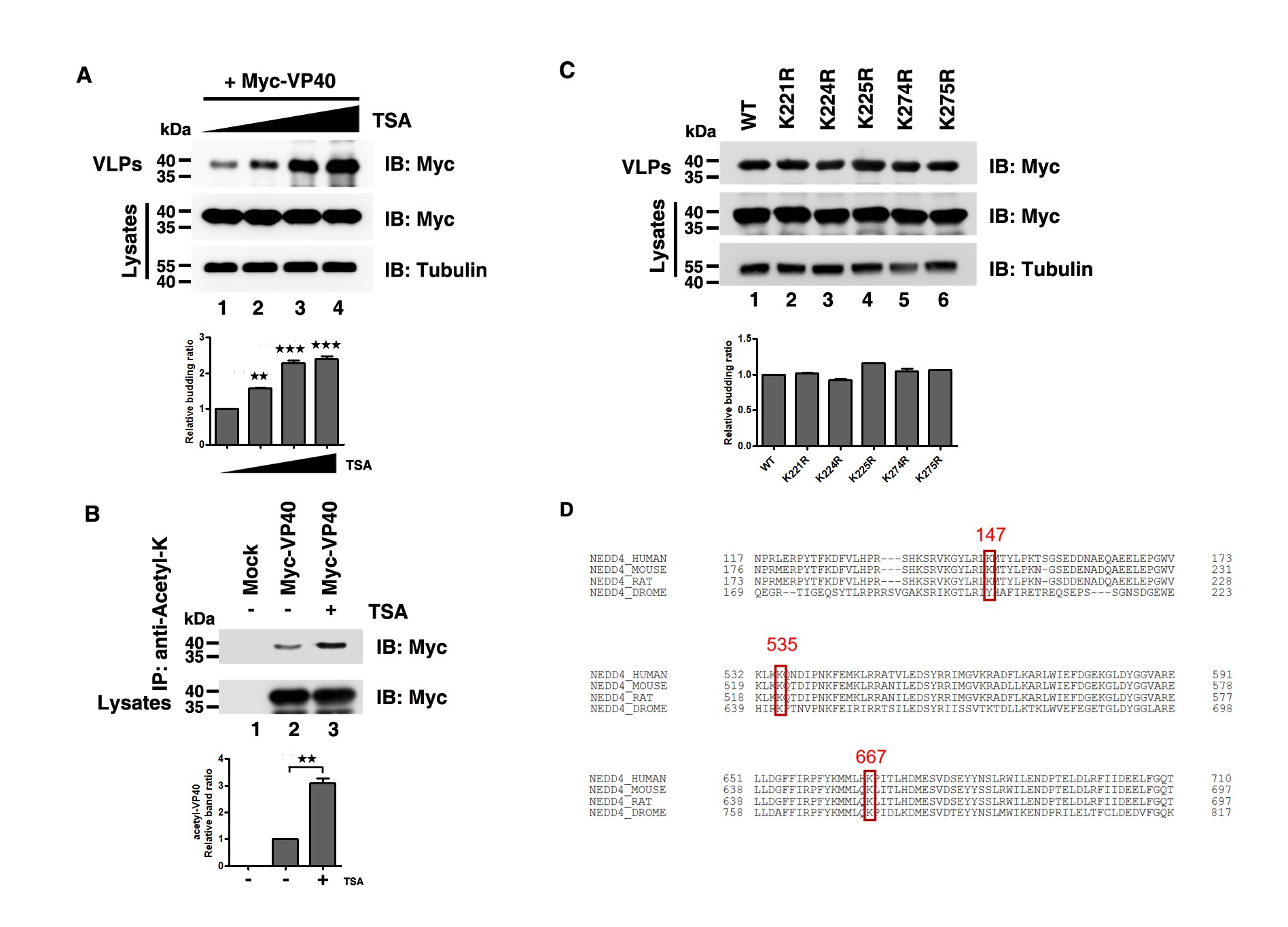

Supplement: S1 Fig — (A) The release of VP40 VLPs was measured after adding TSA. (B) HEK293T cells were transfected with vectors or Myc-VP40 immunoprecipitated with an anti-Acetyl-K antibody and analyzed via immunoblotting with an anti-Myc antibody to detect the acetylation of VP40. (C) Measurement of the release of VP40 and mutant VLPs. (D) The conserved sites are K535 and K667. Error bars, mean ± SD of three experiments. Student’s t test; *p < 0.05; **p < 0.01; ***p < 0.001. (TIF) [file ppat.1009616.s001.tif]

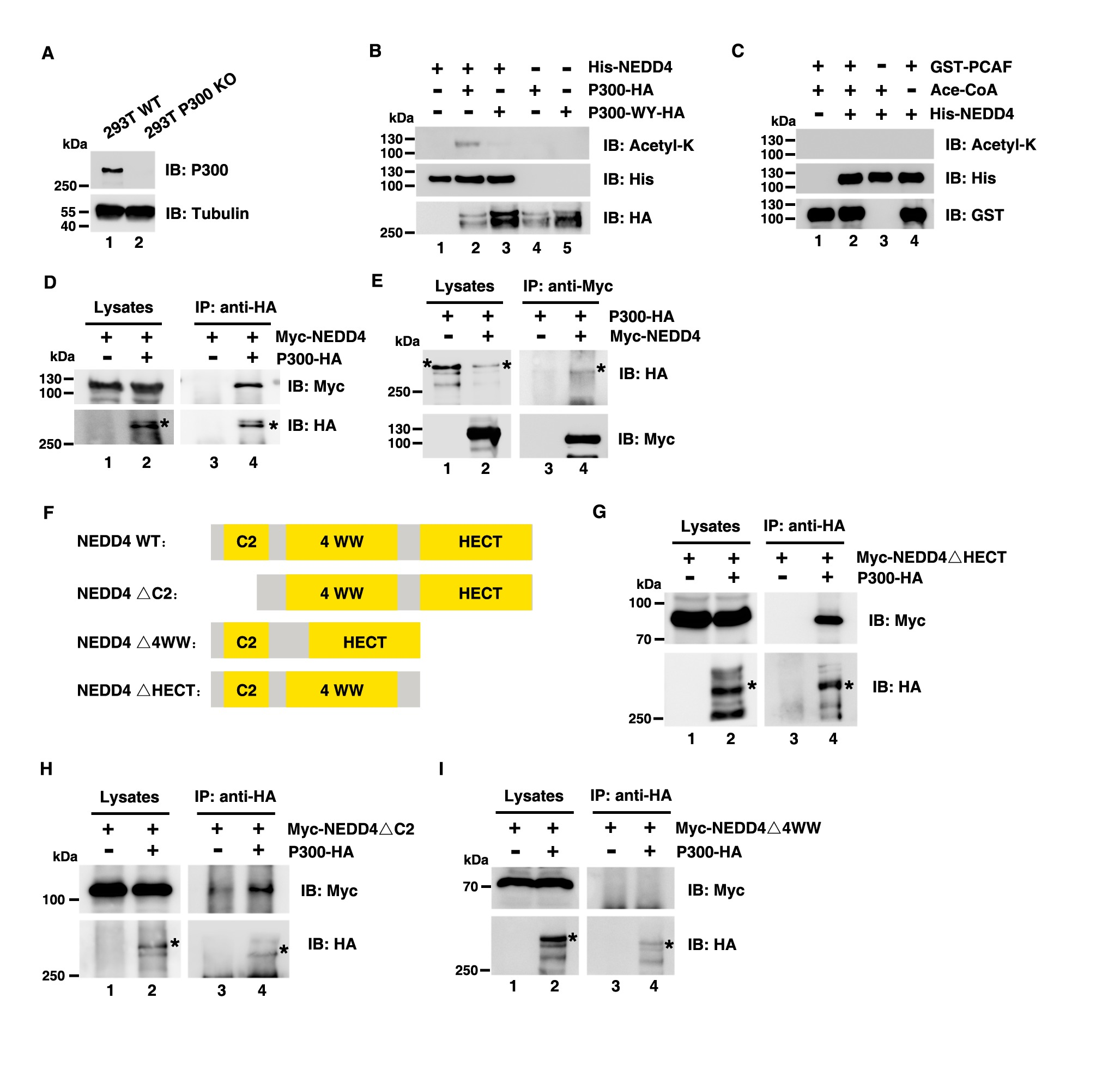

Supplement: S2 Fig — (A) HEK293T P300 knockout cells were analyzed using immunoblotting. (B) In vitro acetylation assays were used to measure purified His-NEDD4 that was incubated with P300-HA and P300-WY-HA, which were immunoprecipitated from HEK293T cells and then analyzed using immunoblotting with an anti-Acetyl-K antibody for detecting the acetylation of NEDD4. (C) An in vitro acetylation assay was used to measure purified His-NEDD4 that was incubated with purified GST-PCAF followed by detection of NEDD4 acetylation. (D) and (E) Interactions between P300-HA and Myc-NED4. The specific band of P300 is indicated by a “*”. (F) Schematic drawing of the NEDD4 WT and mutants. (G)-(I) Interactions between the P300-HA and Myc-NED4 mutants. The specific band of P300 is indicated by a “*”. (TIF) [file ppat.1009616.s002.tif]

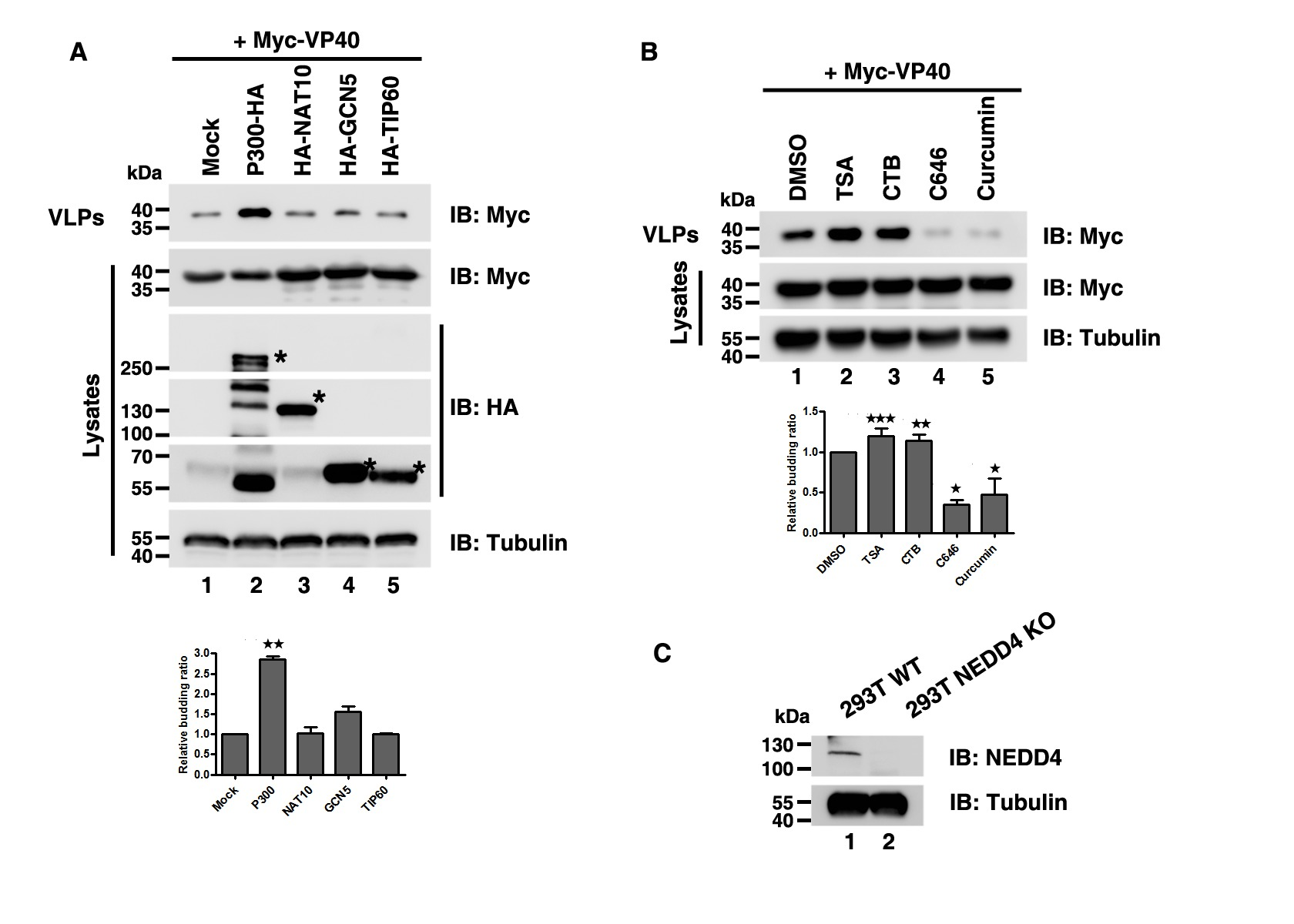

Supplement: S3 Fig — (A) The release of VP40 VLPs was measured when overexpressing acetyltransferases. The target bands of acetyltransferases are indicated by a “*”. (B) The release of VP40 VLPs was measured in response to treatment with P300 activators and inhibitors in the HEK293T WT cell lines. (C) HEK293T NEDD4 knockout cells were analyzed using immunoblotting. Error bars, mean ± SD of three experiments. Student’s t test; *p < 0.05; **p < 0.01; ***p < 0.001. (TIF) [file ppat.1009616.s003.tif]

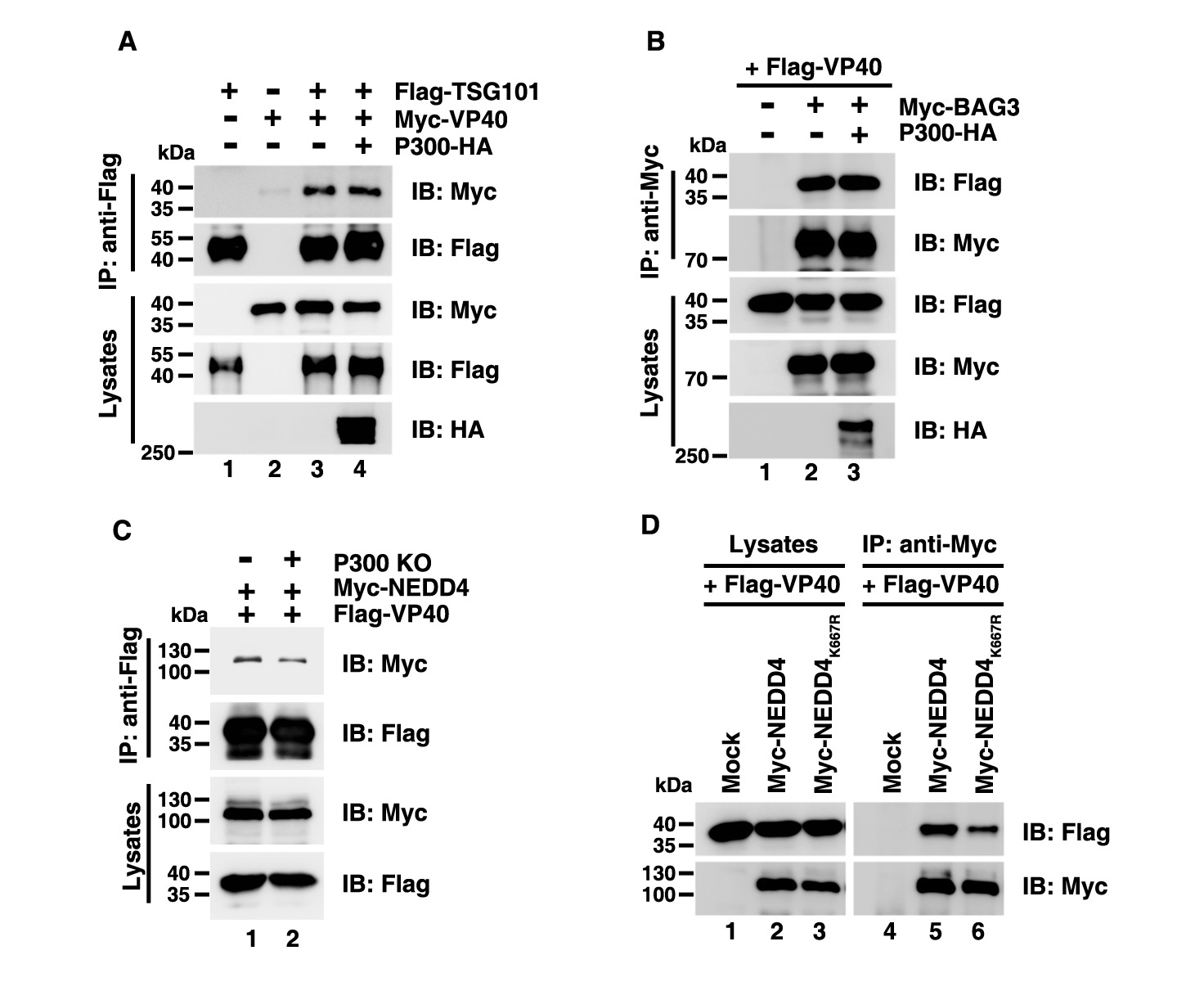

Supplement: S4 Fig — (A)-(B) Interactions were measured by overexpressing the indicated plasmid combinations to detect the influence of P300 on the interactions between VP40 and TSG101(A) or BAG3(B). (C) Interaction between VP40 and NEDD4 in HEK293T P300 KO cell lines.(D) Interaction between VP40 and NEDD4 or NEDD4K667R in HEK293T WT cell lines. (TIF) [file ppat.1009616.s004.tif]

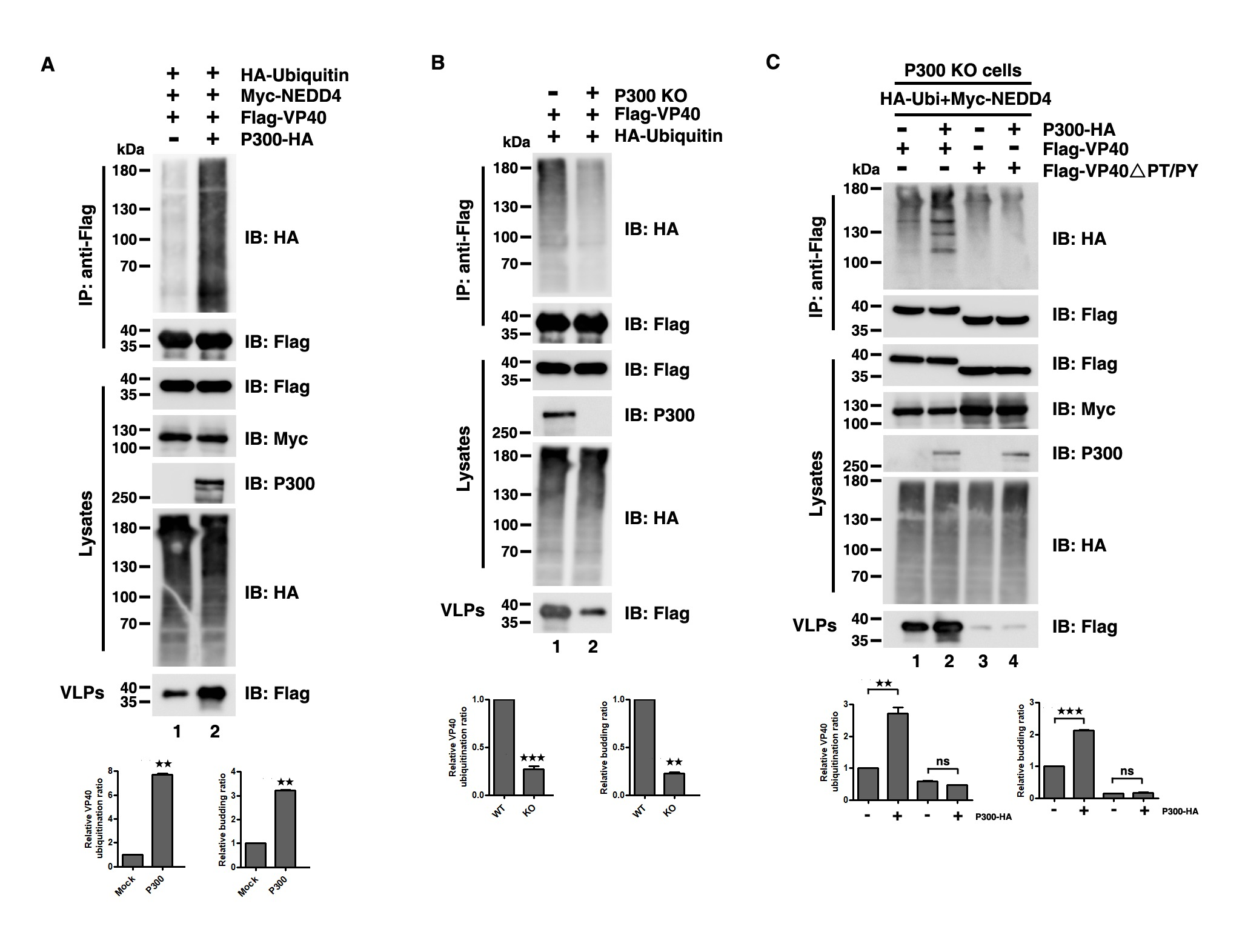

Supplement: S5 Fig — (A)-(C) Cells were transfected with the indicated plasmid combinations to measure the exogenous ubiquitination of VP40 by overexpressing P300-HA and HA-ubiquitin in HEK293T cells (A), Myc-NEDD4 and HA-ubiquitin in P300 KO cells (B), or overexpressing P300-HA when the interaction between VP40 and NEDD4 was impaired in P300 KO cells (C). Error bars, mean ± SD of three experiments. Student’s t test; *p < 0.05; **p < 0.01; ***p < 0.001. (TIF) [file ppat.1009616.s005.tif]
